# Design and Optimization of the Injection Mold for Rubber Stators in Oil Production Single-Screw Pumps

**DOI:** 10.3390/polym17040503

**Published:** 2025-02-14

**Authors:** Haiming Chen, Chongwei Miao, Guangyi Lin, Weimin Yang, Huijin Li

**Affiliations:** 1 College of Mechanical and Electrical Engineering, Qingdao University of Science and Technology, Qingdao 266000, China; gylin666@163.com (G.L.); yangwm@mail.buct.edu.cn (W.Y.); 2 Zhucheng Yongan Rubber Technology Limited Company, Zhucheng 262200, China; 15166051068@163.com; 3 College of Polymer Science and Engineering, Qingdao University of Science and Technology, Qingdao 266000, China

**Keywords:** injection mold, MOLDFLOW analysis, mold flow analysis, orthogonal test

## Abstract

This study aimed to optimize the injection mold for oil production single-screw pump rubber stators. MOLDFLOW2023 analysis post-design determined the optimal gate position and gating system, and it also analyzed the impact of key parameters on quality. The optimization led to a significant improvement in product quality. The most influential factors were mold temperature, melt temperature, and injection time. The best settings were a mold temperature of 100 °C, a melt temperature of 70 °C, an injection time of 30 s, a holding time of 10 s, and a holding pressure of 60 MPa. This resulted in a 1.8–2.7% decrease in volume shrinkage and a 1.3–0.9% decrease in sink index, enhancing the quality of rubber stators and advancing injection molding technology.

## 1. Introduction

**Figure 1:**
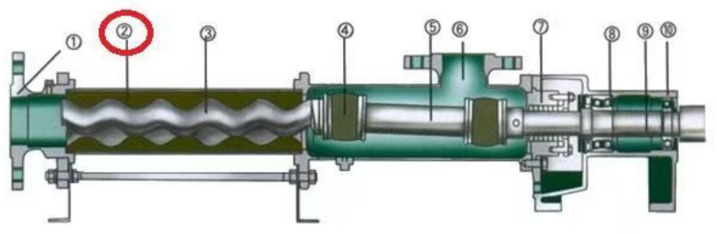
Structural diagram of a single-screw pump. ① Discharge Body. ② Stator. ③ Rotor. ④ Universal Joint. ⑤ Intermediate Shaft. ⑥ Suction Chamber. ⑦ Shaft Seal Element. ⑧ Bearing. ⑨ Drive Shaft. ⑩ Bearing Housing.

Figure 1 shows the structure of a single-screw pump. The stator of this single-screw pump, as shown in Figure 1②, is one of the most important parts of a single-screw pump and is used to realize liquid transfer and increase pressure. The inner cavity of the screw pump stator is spiral. When the screw rotates, the liquid is inhaled, pushed forward along the spiral groove, and then discharged [1].

The efficiency and reliability of oil production equipment are essential for the sustainable development of the industry. Single-screw pumps possess a unique capability to handle viscous fluids and solids, playing a pivotal role within this sector. The rubber stator, a key component of these pumps, is responsible for the transportation of liquids and the enhancement of pressure through its spiral inner cavity. The design and quality of the rubber stator directly impact the pump’s performance, making it a critical area for research and development. However, the complex double helix structure of the stator requires a precise and efficient molding process to ensure consistency in quality and performance. Current injection molds do not fully meet these requirements, leading to issues such as volume shrinkage, sink marks, and inadequate filling, which can compromise the functionality of the stator. A review of the existing literature reveals that, while there is an abundance of research on injection molding processes, there is a scarcity of studies specifically focused on the optimization of molds for rubber stators in single-screw pumps. Therefore, this study aims to fill this gap by designing and optimizing an injection mold for rubber stators used in oil extraction single-screw pumps.

Single-screw pumps play a significant role in oil production, and their performance and reliability directly affect the efficiency of oilfield operations. Research indicates that optimizing the design of single-screw pumps can significantly enhance their recovery rate and operational efficiency [2]. Moreover, the continuous advancement of rubber injection molding technology has provided new possibilities for the manufacture of rubber stators, with advanced injection molding techniques improving product quality and consistency [3]. In terms of mold design, the use of computational fluid dynamics (CFD) and finite element analysis (FEA) technologies can achieve precise optimization of injection molds, thereby increasing molding efficiency and product quality [4].

Product analysis is an important piece of preparatory work before the beginning of design. It is necessary to understand the shape, size, and accuracy requirements of the product in detail. This step ensures that the subsequent design can accurately meet the product requirements [5]. Knowing the specifications of customers for the injection molding machine will determine the range of mold sizes and possible design options. According to the maximum injection volume, clamping force, product accuracy requirements, and the economy, the number of cavities and their arrangement schemes are determined. When selecting the parting surface, the mold structure should be simple and the parting should be easy, without damaging the appearance and function of the plastic parts. A reasonable flow channel and gate can ensure that the rubber can fill the cavity smoothly. The guiding and positioning device ensures the correct installation and operation accuracy of the mold.

The shape of the stator is simple; the outside is a regular cylinder, and the parting surface is selected at the maximum contour. Because the inside is a double helix structure, a specific core-pulling mechanism is also needed to be clamped in the middle of the cavity and extracted during demolding. The mold is arranged by one mold, one cavity, and one gate. In order to ensure the accurate position of the moving mold and the fixed mold when closing the mold, the guiding mechanism must be designed. The guide mechanism is composed of a guide column and a guide sleeve. The guide column is fixed in the moving die, and the guide sleeve is fixed in the fixed die. Generally, four guide columns should control the cooperation between the guide column and the guide sleeve, and the cavity and the template are connected by fastening screws [6].

In short, in the design process, it is also necessary to take into account the technical conditions of mold manufacturing, the mold use environment, production costs, and other factors. The ultimate goal is to design a mold that meets the product requirements and is economical and practical.

The existing screw pump stators are all manufactured by pressure injection molding or secondary pressure transfer molding, and they are rarely manufactured by injection molding. In this study, a method for optimizing the injection mold of a rubber stator in a single-screw pump was introduced. By focusing on the unique double helix structure and combining advanced MOLDFLOW simulation with orthogonal tests, this study provides a new perspective regarding mold design optimization, which is a major element of progress in the existing literature.

List of objectives: to analyze current mold design challenges specific to rubber stators using MOLDFLOW simulations; to design an injection mold that minimizes volume shrinkage and sink marks and enhances fill efficiency; to evaluate the impact of key molding parameters on stator quality through orthogonal testing; to optimize the mold design for improved stator performance and quality; to contribute valuable insights to rubber injection molding technology for future research.

## 2. Materials and Methods

### 2.1. Selection of Rubber-Injection Machine

When choosing a rubber-injection machine, many factors should be considered, such as the shape and size of the mold. It is necessary to ensure that the distance between the rods of the injection machine, the maximum mold thickness, and the minimum mold thickness can meet the size requirements of the mold. The weight of the product is also an important factor in the selection of the injection machine. It should be calculated according to 80% of the maximum injection capacity of the injection machine to ensure a sufficient injection capacity [7].

The following discusses the injection machine parameters that affect the mechanical properties of the rubber stator:

(1) Injection Pressure: This parameter is crucial for ensuring that the rubber is adequately compacted within the mold. Higher injection pressure can increase the density and mechanical strength of the stator.
(2) Mold Temperature: The temperature of the mold affects the vulcanization rate and the final characteristics of the rubber. The optimal mold temperature ensures uniform curing and enhances the stator’s elasticity and resistance to deformation.
(3) Injection Speed: The speed at which the rubber is injected influences the microstructure of the stator, which in turn affects its mechanical properties. A balanced injection speed prevents incomplete filling or excessive heat buildup, both of which can compromise the integrity of the stator.
(4) Curing Time: The duration of exposure to heat and pressure during the curing process is vital for the rubber. An insufficient curing time can result in poor mechanical properties in the stator, while over-curing may lead to brittleness.
(5) Back Pressure: This parameter helps maintain the consistency of the rubber’s flow and prevents material degradation, which could negatively impact the stator’s mechanical properties.
(6) Machine Capacity: The machine’s injection capacity should match the volume of the stator to be produced, ensuring that the machine can handle the required amount of rubber without being overloaded.
(7) Clamping Force: A sufficient clamping force is necessary to keep the mold closed during the injection process, preventing flash and ensuring that the stator is well formed.

### 2.2. Mold Structure Design

#### 2.2.1. Parting Design

There are many choices for the parting surface of each product. The selection of different parting surfaces will directly affect the forming quality of the product, the difficulty of mold manufacturing, the difficulty of demolding and so on. Therefore, when selecting the parting surface, multiple factors need to be considered to ensure the quality of the plastic parts, the simplicity of the mold structure, and the production efficiency. The following is a list of several selection principles:

(1) Easy demolding: The position of the main parting surface should be selected at the maximum contour profile in the demolding direction of the part, that is, the maximum cross-section of the plastic part in this direction. This simplifies the overall design of the mold and improves the manufacturing efficiency of the mold.
(2) Appearance quality: For plastic parts that focus on appearance, the influence of demolding on the appearance surface should be considered when selecting the parting surface.
(3) Conciseness of mold structure: Choosing the correct parting surface not only makes the mold structure simple but also is more conducive to the demolding of plastic parts.

**Figure 2:**
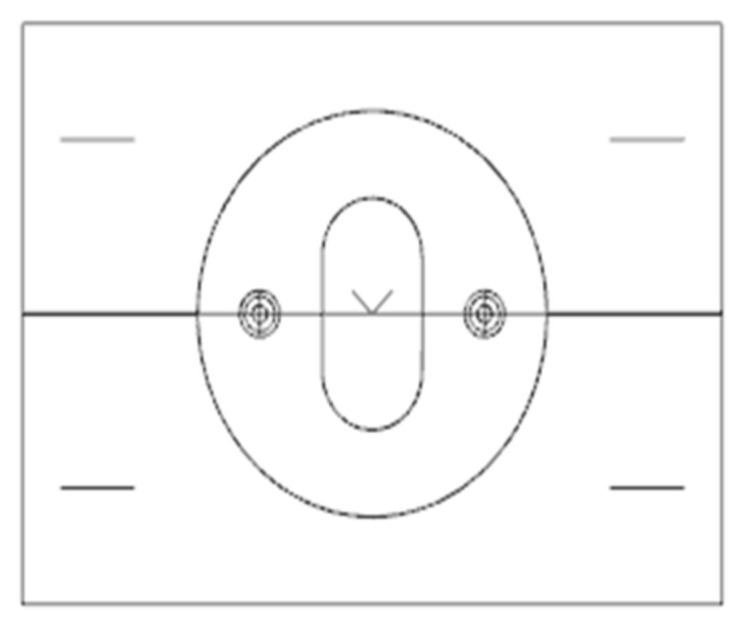
Side view of the classification surface.

**Figure 3:**
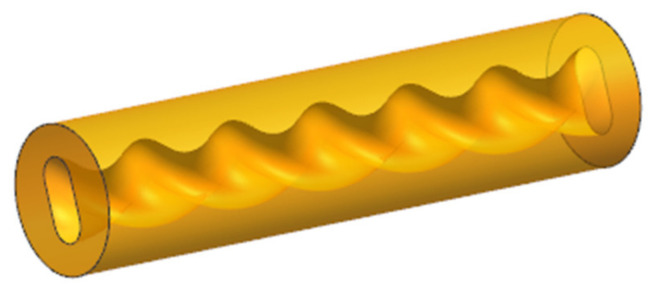
Three-dimensional model of the stator.

The selection principle is only listed in a few parts, but, in general, in actual operation, designers need to make comprehensive judgments and selections according to the specific characteristics of plastic parts and production requirements, combined with the above principles, in order to achieve the best molding effect and economic benefits. In order to facilitate the mold opening, the parting surface is set at the maximum contour section, as shown in Figure 2. The stator structure is simple and the appearance is a cylindrical structure, as shown in Figure 3 [8].

#### 2.2.2. Gating System

The main channel is the first channel for the rubber to pass through during the injection process, ensuring that the rubber can smoothly flow into the mold cavity. The structural design of the main runner has been fixed by the equipment, and the gate diameter is generally between 8 mm and 12 mm.

The split channel ensures the smooth transition of the flow of the rubber and the rapid entry into the cavity. The cross-sectional shape of the flow channel will affect the flow and flow efficiency of the rubber in the flow channel. The cross-sectional shapes of runners are round, rectangular, trapezoidal, U-shaped, and semi-circular. The circular cross-section flow channel has a high flow efficiency, but the cost is high. The semi-circular cross-section flow channel is convenient to manufacture compared with the circular cross-section flow channel [9].

#### 2.2.3. Guiding Agencies

**Figure 4:**
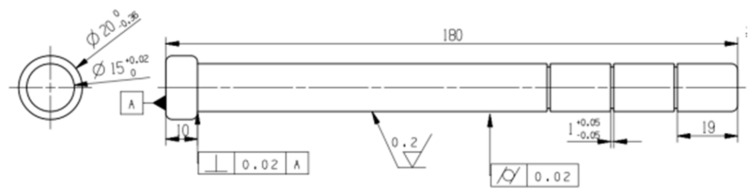
Two-dimensional diagram of the guide column.

The guiding mechanism has a supporting effect on the mold and can accurately locate and successfully complete the whole process of opening and demolding during the movement of the mold. The size of the stator mold is small, and the use of 4 guide pillars as the support body of the mold is proposed, as shown in Figure 4.

#### 2.2.4. Mold Core Structure

**Figure 5:**
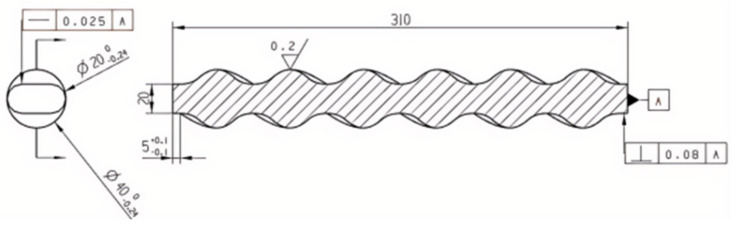
Two-dimensional diagram of the module core.

The core and the cavity cooperate with each other and are located in the internal center. When the rubber fills the cavity, the molded part, which is withdrawn by the spiral movement of the core, retains the double helix structure inside the stator. The total length of the die core is 310 mm. According to the set eccentricity e = 10 mm, pitch t = 100 mm, number of stages K = 3, rotor diameter D = 20 mm, use UG to draw the core structure by sketching, sweeping, and helical lines. The two-dimensional diagram is shown in Figure 5 [10].

#### 2.2.5. Template Structure Design

**Figure 6:**
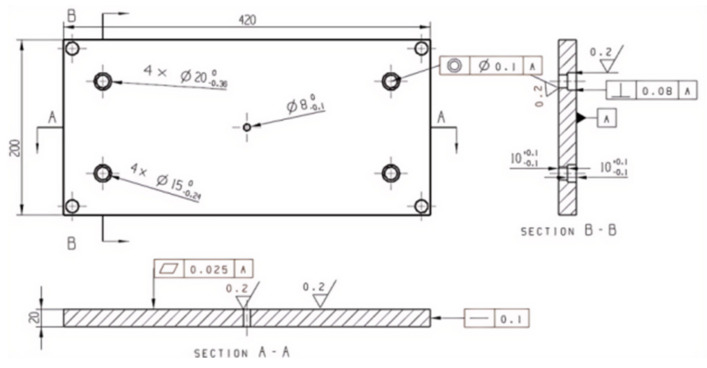
Upper mold seat.

**Figure 7:**
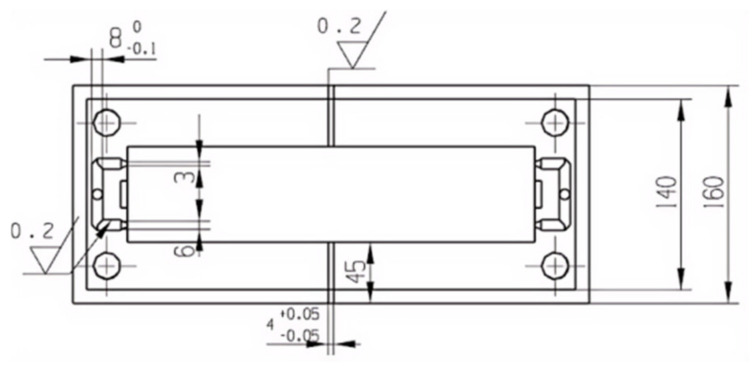
Moving mode.

**Figure 8:**
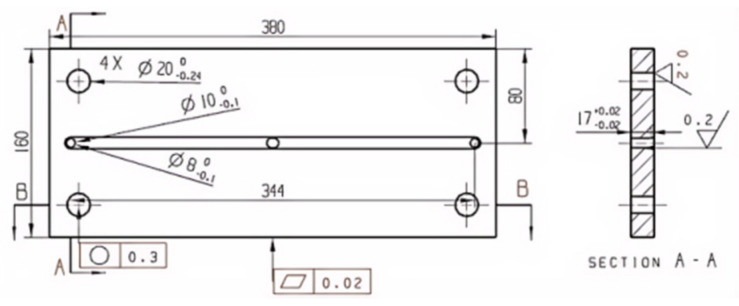
Middle template.

The upper template is utilized mainly to fix the mold so that the whole mold is connected with the injection machine. Usually, there is an injection hole in the upper mold seat, the size of which corresponds to the nozzle of the injection machine, and the rubber is first entered into the mold through the injection hole. The lower die seat is associated with the upper die seat, and the function is consistent. It is fixed at the bottom of the injection machine, mainly supporting the mold, as shown in Figure 6. The moving mold and the fixed mold constitute the main structure of the mold, as shown in Figure 7. The moving mold completes the opening and closing operation of the moving part of the mold, and the fixed mold is fixed on the injection machine to provide support and positioning for the mold to ensure the stability of each opening and closing process. The stator mold cavity is provided by both the fixed mold and the moving mold. The mold cavity is a cylindrical structure, in the middle of which is the core mold, which together constitutes the stator forming structure. In order to ensure the stability of the mold cavity and the moving mold, screws are used to fix it. The role of the middle template is mainly to set the first-stage shunt channel of the gating system. Because the stator flow channel adopts a multi-stage flow channel, in order to make it convenient for processing, a semi-circular flow channel is adopted on the middle template, as shown in Figure 8. A cold material well is arranged in the middle position of the first-stage shunt channel to prevent the cold material from entering the cavity during injection [11].

### 2.3. Finite Element Analysis of a Rubber Stator Injection Molding Process

#### 2.3.1. Establishment of a Finite Element Model

**Figure 9:**
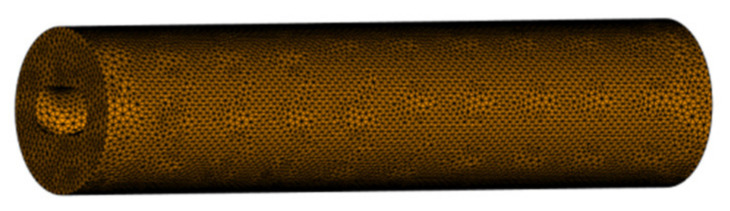
Grid division of double-sided stator layers.

The model is established according to the parameters, and then the grid is divided. The stator model is divided by a double-layer grid, and the appropriate grid density should be set when dividing the grid. The more the number of grids is divided, the more accurate the simulation results are and the longer the simulation time is in the analysis. If the mesh is too sparse, the results of the analysis will not be accurate enough to meet the purpose of the simulation analysis. Set the grid side length, click to divide the grid immediately, and obtain the divided model, as shown in Figure 9.

#### 2.3.2. Establishment of Flow Channel

After importing the model into the software, the model is meshed first, and then the flow channel nodes are manually created according to the coordinate points. In order to ensure that the gate position is consistent with the model, the two ends of the gate are connected by a straight line and the igs format is derived. In the second step, it is imported into MOLDFLOW, and the gate grid can be obtained by clicking ‘generate grid’ in the ‘grid’ tab. The third step is to use coordinates to construct flow channel nodes, define cylinder attributes, and divide grids. The overall parameters of the gating system are shown in Table 1.

**Figure 10:**
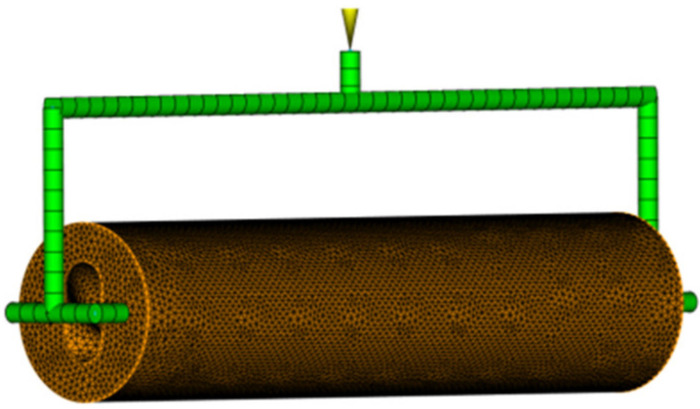
Flow channel.

After the establishment of the flow channel is completed, as shown in Figure 10, a connectivity diagnosis is required. In the inspection report, the connected area shows red and the unconnected area shows blue.

#### 2.3.3. Orthogonal Test Factors and Levels

Nitrile rubber of grade Generic NBR 1 from Generic was selected as the mold filling material for the stator of the progressive cavity pump. This study chose nitrile rubber (NBR) due to its unique properties: Oil Resistance: NBR is renowned for its resistance to oil and chemicals, making it an ideal material for single-screw pumps in oil production; Toughness and Durability: NBR has excellent toughness and wear resistance, which are crucial for withstanding wear in industrial applications; Wide Operating Temperature Range: This is essential for the reliability and service life of the rubber stator under varying environmental conditions.

According to the material library, the screw pump stator with a melt temperature range of 70~100 °C and a mold temperature range of 80~200 °C can be found. This melt temperature range ensures that the nitrile rubber is fully melted during injection molding without causing thermal degradation. The lower limit ensures flowability, while the upper limit prevents excessive degradation that could affect the performance of the rubber. This mold temperature range promotes proper curing and controls the cooling speed of the rubber. Lower temperatures help avoid internal stress caused by rapid cooling, while higher temperatures aid in achieving uniform curing. Although the shape of the screw pump stator is simple, the internal double helix structure causes the wall thickness to be uneven, and the rubber is difficult to fill during mold filling. Therefore, it is necessary to set the holding pressure. After the filling is completed, the injection machine continues to provide pressure to fill the cavity with the rubber. The holding pressure should select the appropriate value. If the holding pressure is too high, the required clamping force will increase, which will bring a burden to the injection molding machine. If the holding pressure is too low, it will make it difficult for the rubber to fill the mold cavity. According to the injection pressure, to obtain a pressure-holding pressure interval of 50~90 Mpa, a pressure-holding time interval of 8~16 s is required. Holding pressure time refers to the duration for which pressure is maintained to fill the cavity with rubber and carry out curing. This range is chosen to achieve a balance, ensuring the complete filling of the rubber and avoiding over-curing, which could lead to material degradation or dimensional instability. According to the stator volume and control of the flow rate of the injector derived from the approximate injection time of about 30 s, the injection time interval will be set as 30~50 s. Because the injection time is too short, it will lead to a surge in flow rate, causing damage to the mold and injection machine. The longer the injection time, the more controllable the filling becomes. In accordance with the parameter range, we can establish a factor level table, as shown in Table 2 [12].

## 3. Results and Discussion

### 3.1. The Effect of Process Parameters on Mechanical Properties

Mold temperature significantly affects a product’s thermal stability and long-term performance by influencing material expansion and contraction, which can impact dimensional stability, internal stress distribution, and mechanical properties like tensile strength and toughness. Higher mold temperatures can lead to greater internal stresses post-cooling, reducing impact resistance.

Melt temperature directly impacts the flowability and curing speed of the material. Excessive melt temperatures may cause material degradation, affecting molecular structure and thus mechanical strength and durability. The proper melt temperature ensures uniform molecular alignment, enhancing mechanical performance.

Injection time dictates the speed at which the material fills the mold, with improper speeds leading to uneven material distribution and affecting mechanical properties. The optimal injection time reduces internal bubbles and voids, increasing product density and strength.

Holding pressure helps compact material in the mold, reducing voids. An inappropriate holding pressure can cause uneven internal stresses, affecting mechanical properties. Proper holding pressure improves product density, enhancing compressive and wear resistance.

Holding time determines how long material remains compacted in the mold. Too short a time may prevent full mold filling, while too long a time can affect dimensional stability. The appropriate holding time allows for complete mold filling, reducing internal defects and improving tensile and fatigue strength.

### 3.2. Analysis of Orthogonal Experimental Results

This experiment mainly analyzes the stator injection molding process. Through MOLDFLOW simulations, the effects of mold temperature, melt temperature, injection time, holding pressure, and holding time on volume shrinkage rate and sink index are explored. The influence degree of each factor on the result is determined by orthogonal testing, and then the optimization is carried out to find the optimal solution to improve the quality of the stator. A filling + packing analysis was performed according to the experimental variables in the orthogonal experimental factor level table. The experimental results are shown in Table 3.

### 3.3. Range and Variance Analysis

According to the data in the table, the extreme values of volumetric shrinkage rate and sink index were calculated as shown in Table 4 and Table 5, respectively, where K1, K2, K3, K4, and K5 (the sum of all the levels under each factor), k1, k2, k3, k4, and k5 (the total sum divided by the number of levels yields the mean value of each level for each factor), and the maximum mean minus the minimum mean value for each factor is the extreme value R.

**Figure 11:**
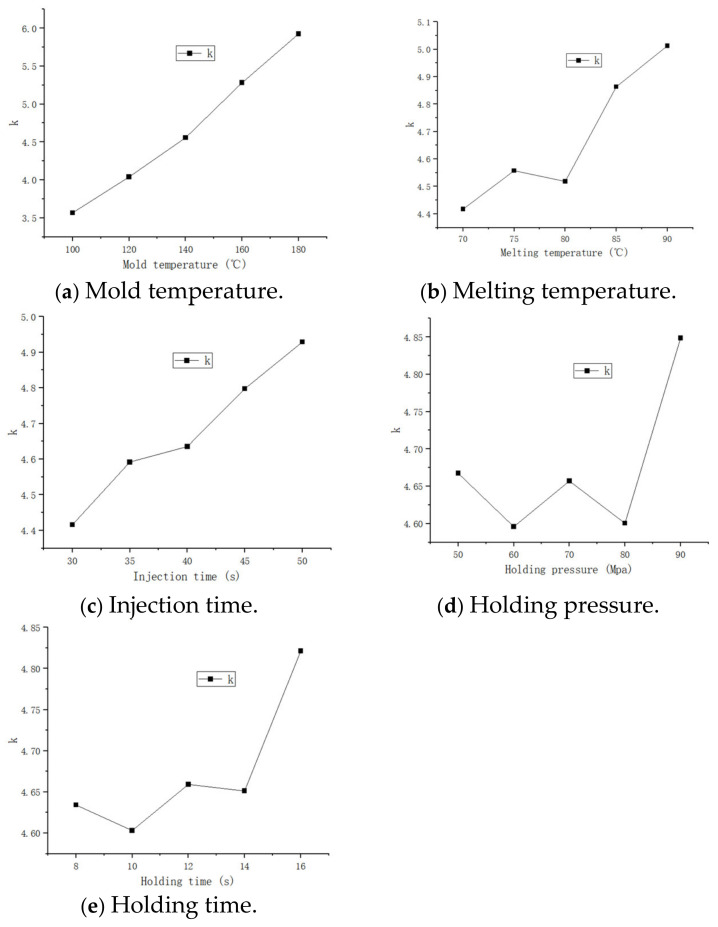
Fold line diagram of influencing factors of volume shrinkage.

The range analysis results show that R (Mold temperature) > R (Melting temperature) > R (Injection time)> R (Holding pressure) > R (Holding time). Among the five factors, the mold temperature has the greatest influence on the quality index, followed by the melt temperature and injection time, while the other two ranges account for a small proportion and have little effect on the results. The level of each factor is summarized, and the line chart of each factor is established according to the range table, as shown in Figure 11. The line chart can directly see the influence relationship between each factor and quality index. Among them, the volume shrinkage rate is positively correlated with the mold temperature. The higher the mold temperature, the higher the volume shrinkage rate. This is because the thermal expansion rate of the rubber material mainly depends on the temperature. At the beginning of the injection molding, the mold temperature is basically maintained at a high temperature, and the temperature decreases after the parts are solidified and cooled, forming a large temperature difference. Due to the mechanism of thermal expansion and cold contraction, the volume shrinkage rate varies greatly.

It can be seen from the line chart that the melt temperature is basically positively correlated with the quality index. The reason is that, the higher the melt temperature is, the larger the volume will expand during the injection process. After the injection is completed, the product will shrink when it cools to the ambient temperature, the density will decrease, and the volume will naturally shrink to a greater degree [13].

It can be seen from the line chart that the trend of injection time is increasing. If the injection time is too long, the rubber will be filled slowly. After continuously absorbing the mold temperature, the melt temperature will increase, resulting in an increase in volume shrinkage. Therefore, it is very important to choose the appropriate injection time.

It can be seen from the holding time and holding pressure curve that there is no obvious positive and negative correlation trend. It is difficult to express simply by relying on a simple linear relationship. Because the range is very small, the influence can be ignored, but it should also be selected according to the actual situation.

The condition corresponding to the minimum value of the mean value of each level of each factor in the range result is the optimal solution of the minimum volume shrinkage rate, that is, the mold temperature is 100 °C, the melt temperature is 70 °C, the injection time is 30 s, the holding time is 10 s, and the holding pressure is 60 MPa.

We calculate the mean value of each level for each factor, which in turn leads to a table of the range of the sink index, as shown in Table 5.

**Figure 12:**
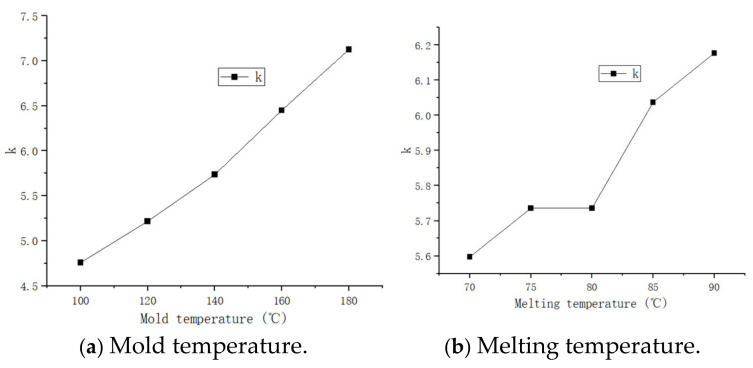

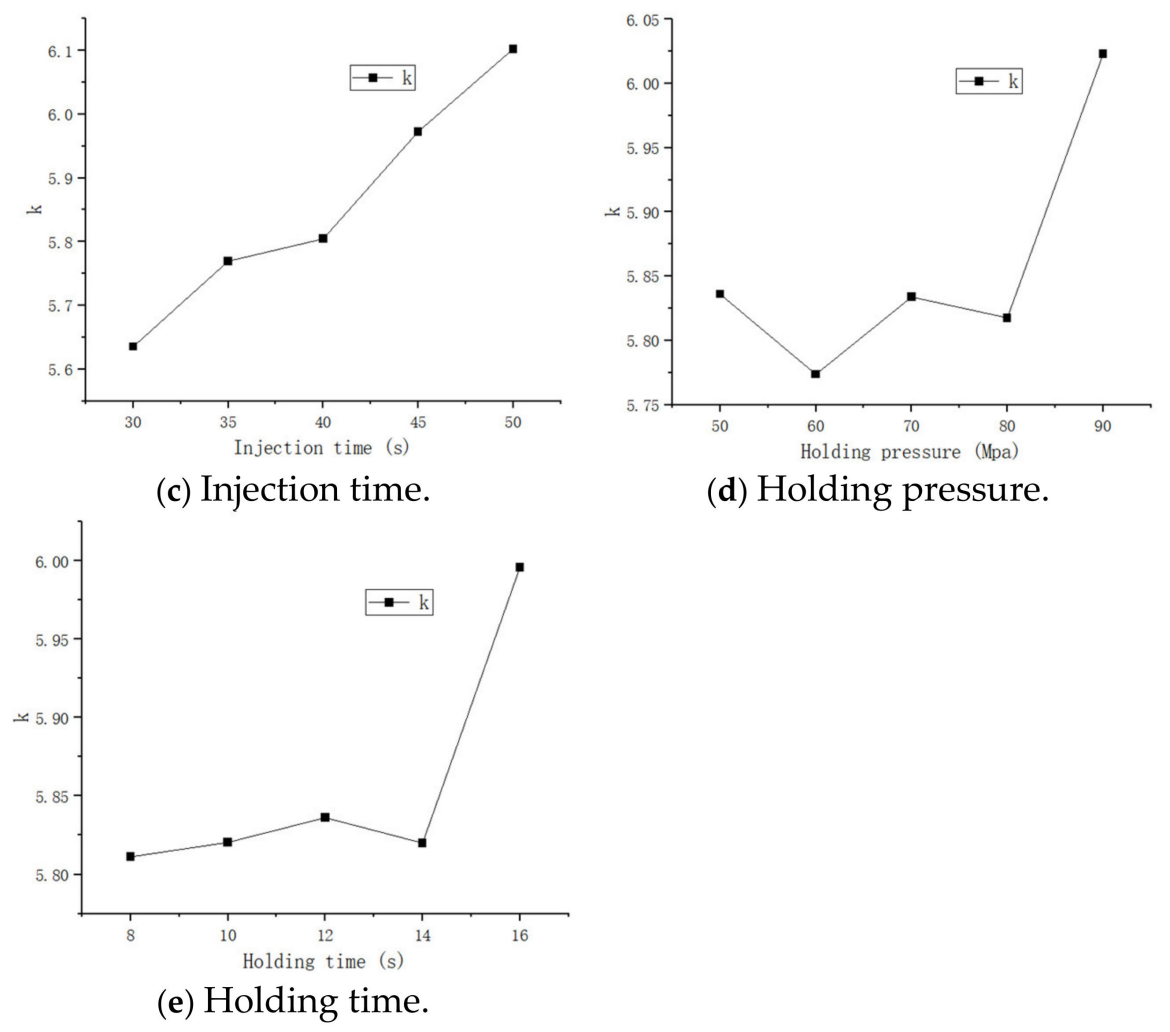
Fold line diagram of the influencing factors of the sink index.

The range analysis results show that R (Mold temperature) > R (Melting temperature) > R (Injection time)> R (Holding pressure) > R (Holding time), and the sink index is most affected by the mold temperature, followed by the melt temperature; the remaining results are basically consistent with the volume shrinkage rate results. The influence trend diagram of each factor generated by the software is shown in Figure 12.

It can be seen from Figure 12 that the mold temperature has the greatest influence on the sink index within the parameter range, followed by the melt temperature and injection time, while the other two factors have less influence. At the beginning of the injection molding, the mold temperature is basically maintained at a high temperature and the temperature of the part is reduced after curing and cooling. The volume change caused by the thermal expansion of the product is particularly obvious, and the volume shrinkage makes the sink index increase.

The comparison trend diagram shows that the melt temperature is also basically positively correlated with the quality index. The reason is that, the higher the melt temperature is, the more the volume will expand during the injection molding process. After the injection is completed, the product will be cooled to the ambient temperature. The parts will shrink seriously, the density will decrease, the volume will naturally shrink, and the sink index will be larger.

The trend of injection time is increasing. The long injection time will increase the temperature difference generated by the rubber in the filling process, thereby increasing the internal stress caused by the temperature difference, thereby increasing the possibility of shrinkage. Therefore, the appropriate injection time can be selected to ensure quality [14].

By comparing the trend chart, it is found that the holding pressure and holding time can maintain a low sink index in some intervals. There is no accurate positive and negative correlation trend, because their relationship with the sink index is complex and a single linear relationship is difficult to express. It can only be selected from a reasonable interval. If the trend increases suddenly at a certain time point, a large internal stress will be generated under long-term pressure, which will affect the sink index [15].

Combined with the range results of volume shrinkage and sink index, the minimum value of the average value of each level of each factor is determined and the optimal injection parameter table is obtained, as shown in Table 6.

According to the range analysis results, the injection parameters of the stator are obtained and the influence of each factor on the results is determined. In order to verify the accuracy of the results, an analysis of variance was established, and the analysis results are shown in Table 7 and Table 8.

According to the variance analysis results, the mold temperature and melt temperature are the main influencing factors, followed by the injection time. The remaining two *p* values are much larger than the other items, and the reference significance is small. The *p* value of less than 0.05 is statistically significant and has reference significance [16].

### 3.4. Optimization Results

**Figure 13:**
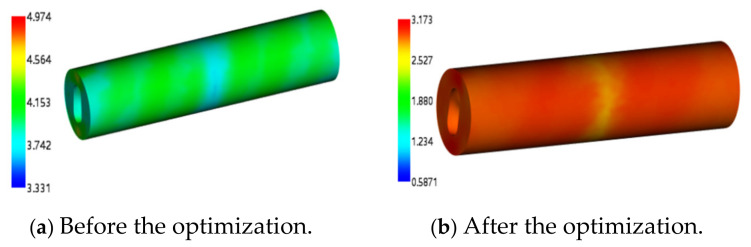
The cloud diagram before and after volume shrinkage optimization.

**Figure 14:**
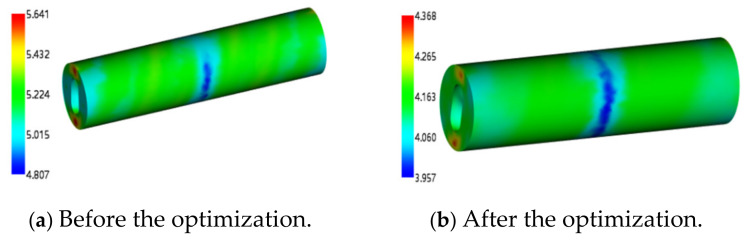
The cloud diagram before and after sink index optimization.

Before optimization, the recommended parameters of the material library were used to obtain the maximum volume shrinkage rate of 4.974% and the minimum volume shrinkage rate of 3.331%, as shown in Figure 13a. The maximum value of the sink index is 5.6415%, and the minimum value is 4.807%, as shown in Figure 14a. Using the optimal solution parameters, the maximum volume shrinkage rate after optimization is 3.173% and the minimum value is 0.5871%, as shown in Figure 13b. The maximum value of the sink index is 4.368%, and the minimum value is 3.957%, as shown in Figure 14b.

Compared with the volume shrinkage rate, it is reduced by 1.801~2.7439%, and the sink index is reduced by 1.2735~0.85%. The main reason is to optimize the mold temperature and melt temperature. If the two temperatures are large, the volume of the rubber stator will expand rapidly and then shrink with the decrease in temperature after the injection molding. In simple terms, with the increase in temperature, the volume expansion trend is obvious, and the shrinkage phenomenon will be more significant. The reasonable setting of these two temperatures can effectively reduce the volume shrinkage and sink index, so as to improve the quality of the stator [17].

## 4. Conclusions

(1) The study suggests that setting the mold temperature to 100 °C, the melt temperature to 70 °C, the injection time to 30 s, the holding time to 10 s, and the holding pressure to 60 MPa can significantly improve the injection mold for rubber stators.
(2) Through optimization, the volume shrinkage rate (reduced from 1.801% to 2.7439%) and the sink index (reduced from 1.2735% to 0.85%) were notably decreased, thereby endowing the stators with excellent mechanical properties and dimensional stability.
(3) The redesigned injection mold has demonstrated a significant improvement in the efficiency of rubber stator production. The research findings hold significant value for the industrial application of rubber injection molding technology, particularly in the oil production sector, where the reliability and quality of stators are paramount.
(4) Future research can further explore the optimization of mold designs for a variety of materials and complex structures, potentially expanding the industrial application scope of this technology.

## Figures and Tables

**Table 1:** Casting system parameters.

	Cold Gate	Cold Shunt	Cold Main Channel
**Cross-sectional shape**	Circular (cone)	Circular (non-cone)	Semi-circular (non-cone)
**Diameter/mm**	3–6 (beginning-end)	8	10 × 8 (diameter × height)

**Table 2:** Table of orthogonal experimental factors.

Factor Level	Mold Temperature/°C	Melting Temperature/°C	Injection Time/s	Holding Pressure/Mpa	Holding Time/s
1	100	70	30	50	8
2	120	75	35	60	10
3	140	80	40	70	12
4	160	85	45	80	14
5	180	90	50	90	16

**Table 3:** Table of results of orthogonal experiments.

Numbering	Mold Temperature/°C	Melting Temperature/°C	Injection Time/s	Holding Pressure/MPa	Holding Time/s	Blank	Volume Shrinkage/%	Sink Mark Index/%
1	1	1	1	1	1	1	3.174	4.369
2	1	2	3	4	5	2	3.394	4.587
3	1	3	5	2	4	3	3.54	4.731
4	1	4	2	5	3	4	3.83	5.017
5	1	5	4	3	2	5	3.896	5.082
6	2	1	5	4	3	5	3.904	5.090
7	2	2	2	2	2	1	3.861	5.048
8	2	3	4	5	1	2	4.051	5.235
9	2	4	1	3	5	3	4.076	5.260
10	2	5	3	1	4	4	4.307	5.448
11	3	1	4	2	5	4	4.489	5.668
12	3	2	1	5	4	5	4.288	5.469
13	3	3	3	3	3	1	4.499	5.678
14	3	4	5	1	2	2	4.832	6.007
15	3	5	2	4	1	3	4.675	5.852
16	4	1	3	5	2	3	5.064	6.236
17	4	2	5	3	1	4	5.359	6.527
18	4	3	2	1	5	5	5.138	6.308
19	4	4	4	4	4	1	5.666	6.830
20	4	5	1	2	3	2	5.177	6.348
21	5	1	2	3	4	2	5.455	6.622
22	5	2	4	1	3	3	5.886	7.048
23	5	3	1	4	2	4	5.363	6.729
24	5	4	3	2	1	5	5.912	7.073
25	5	5	5	5	5	1	7.009	8.157

**Table 4:** Table of range of volume shrinkage.

Level	Mold Temperature	Melting Temperature	Injection Time	Holding Pressure	Holding Time	Blank
k1	3.5668	4.4172	4.4156	4.6674	4.6342	4.8418
k2	4.0398	4.5576	4.5918	4.5958	4.6032	4.5818
k3	4.5566	4.5182	4.6352	4.657	4.6592	4.6482
k4	5.2808	4.8632	4.7976	4.6004	4.6512	4.6696
k5	5.925	5.0128	4.9288	4.8484	4.8212	4.6276
R	2.3582	0.5956	0.5132	0.2526	0.218	0.26

**Table 5:** Table of the range of sink index.

Level	Mold Temperature	Melting Temperature	Injection Time	Holding Pressure	Holding Time	Blank
k1	4.7572	5.597	5.635	5.836	5.8112	6.0164
k2	5.2162	5.7358	5.7694	5.7736	5.8204	5.7598
k3	5.7348	5.7362	5.8044	5.8338	5.8362	5.8254
k4	6.4498	6.0374	5.9726	5.8176	5.82	5.8778
k5	7.1258	6.1774	6.1024	6.0228	5.996	5.8044
R	2.3686	0.5804	0.4674	0.2492	0.1848	0.118

**Table 6:** Optimal parameters.

Mold Temperature/°C	Melting Temperature/°C	Injection Time/s	Holding Time/s	Holding Pressure/MPa
100	70	30	8–10	60

**Table 7:** Analysis of variance of the volume shrinkage rate.

Source	Sum of Squares of Deviations	Degree of Freedom	Mean Square	F Ratio	*p* Value	Significance
A	17.8754564	4	4.4688641	90.5190898	0.000355568	√
B	1.2717536	4	0.3179384	6.440002188	0.049340626	√
C	0.7761632	4	0.1940408	3.93039399	0.106724911	
D	0.2113996	4	0.0528499	1.070501303	0.474472104	
E	0.145016	4	0.036254	0.734343003	0.613982361	
F error	0.1974772	4	0.0493693			

**Table 8:** Analysis of variance of the sink index.

Source	Sum of Squares of Deviations	Degree of Freedom	Mean Square	F Ratio	*p* Value	Significance
A	17.98191256	4	4.49547814	92.08773235	0.00343728	√
B	1.16041056	4	0.29010264	5.942614653	0.034782979	√
C	0.66654376	4	0.16663594	3.413458005	0.130750623	
D	0.18488256	4	0.04622064	0.946807835	0.520486971	
E	0.12279776	4	0.03069944	0.628863433	0.667930271	
F error	0.19526936	4	0.04881734			

## Data Availability

The raw data supporting the conclusions of this article will be made available by the authors upon request.
